# Risk of African swine fever virus introduction into the United States through smuggling of pork in air passenger luggage

**DOI:** 10.1038/s41598-019-50403-w

**Published:** 2019-10-08

**Authors:** Cristina Jurado, Lina Mur, María Sol Pérez Aguirreburualde, Estefanía Cadenas-Fernández, Beatriz Martínez-López, José Manuel Sánchez-Vizcaíno, Andrés Perez

**Affiliations:** 1 VISAVET Health Surveillance Centre and Animal Health Department, Complutense University of Madrid, Madrid, Spain; 2 Department of Diagnosis Medicine/Pathobiology, College of Veterinary Medicine, Kansas State University, Manhattan, Kansas USA; 3 Center for Animal Health and Food Safety, College of Veterinary Medicine, University of Minnesota, Saint Paul, Minnesota USA; 4 Center for Animal Disease Modeling and Surveillance; and Department of Medicine & Epidemiology, School of Veterinary Medicine, University of California, Davis, California USA

**Keywords:** Computational biology and bioinformatics, Computational models

## Abstract

African swine fever causes substantial economic losses in the swine industry in affected countries. Traditionally confined to Africa with only occasional incursions into other regions, ASF began spreading into Caucasian countries and Eastern Europe in 2007, followed by Western Europe and Asia in 2018. Such a dramatic change in the global epidemiology of ASF has resulted in concerns that the disease may continue to spread into disease-free regions such as the US. In this study, we estimated the risk of introduction of ASF virus into the US through smuggling of pork in air passenger luggage. Results suggest that the mean risk of ASFV introduction into the US via this route has increased by 183.33% from the risk estimated before the disease had spread into Western Europe or Asia. Most of the risk (67.68%) was associated with flights originating from China and Hong Kong, followed by the Russian Federation (26.92%). Five US airports accounted for >90% of the risk. Results here will help to inform decisions related to the design of ASF virus surveillance strategies in the US.

## Introduction

The US is the world’s third largest pig producer, with over 11.5 million tons of pork produced per year, and the world’s second largest pork exporter, with exports in 2017 valued at 4.6 billion USD^1^. The introduction of a foreign animal disease (FAD) into the US may have far-reaching economic consequences for the country, due to the emergency response actions required to control the disease, such as herd depopulation and movement restrictions^2^. Preparedness and prevention measures to avoid the introduction of FADs into the US include strict regulations on imports of live animals and animal products, checking of waste containing products that originated overseas (e.g. waste from international flights), application of thermal treatment to inactivate pathogenic microorganisms that may contaminate swill feed, restrictions on animal consumption of animal-derived by-products, and detection systems to facilitate rapid diagnosis of FAD through the country’s national animal health laboratory network^3,4^.

African swine fever (ASF), caused by infection with the ASF virus (ASFV), is one of the most feared FAD in the US. ASF has traditionally been endemic to sub-Saharan Africa and the Italian island of Sardinia, with sporadic epidemics affecting a number of countries through the 20th century. However, in 2007, the ASFV spread into Georgia, Armenia, and Azerbaijan, and subsequently into the Russian Federation, Ukraine and Belarus. In 2014, four European Union countries became infected (Lithuania, Poland, Latvia, and Estonia). Despite prevention and control measures, ASF continued to spread and eight more European countries (Moldova, Czech Republic, Romania, Hungary, Bulgaria, Belgium, Serbia and Slovakia) reported the disease between 2014 and 2019^5^. In August 2018, China officially reported cases on a domestic pig farm^6^, and as of August 2019, 32 Chinese provinces have been affected by the disease and more than 1,170,000 animals slaughtered^7^. ASF has also been reported in Mongolia^8^, Vietnam^9^, Cambodia^10^, North Korea^11^, Laos^12^ and Myanmar^5^.

The recent ASF spread through Europe and Asia has raised concerns among US swine producers that ASFV-contaminated pork products may be illegally introduced into the country, which may infect susceptible animals, resulting in an epidemic in the country. According to the Agricultural Quarantine Activity Work Accomplishment database of the Animal and Plant Health Inspection Service of the US Department of Agriculture, screening activities conducted between 2010 and 2015 resulted in the confiscation of an average of 8,000 pork products per year. Nearly half (45%) of those prohibited pork products were intercepted at international airports inside air passengers’ personal luggage. We refer henceforth to prohibited pork products carried in air passenger luggage as PSPAP.

It is unclear to what extent PSPAP pose a risk of bringing ASFV to US airports prior to customs inspection because seized PSPAP is not routinely diagnosed. The risk is likely greater than nil, given that ASFV has been detected in prohibited agricultural products seized at airports in South Korea, Japan, Taiwan, Thailand, Australia, the Philippines, and Northern Ireland^13–19^. In March-2019, the largest known illegal shipment of pork (1 million pounds) arrived from China at Newark, New Jersey port, one of the busiest entry ports into the US. Prior to the ASF spread in Western Europe and Asia, our analysis of data from July 2016 suggested that the annual average risk of ASFV introduction into the US via PSPAP was 0.06 (95% CI 0.01–0.21). In other words, ASFV could be expected to enter the US illegally in PSPAP once every 17 years on average^20^.

It is unclear how much the risk has changed as a result of the spread of the disease through Europe and Asia in 2018 and 2019. The study here was aimed at i) quantifying the probability of arrival of ASFV-contaminated PSPAP at US airports (before customs inspection), ii) comparing the risk of ASFV introduction into the US via PSPAP (after customs inspection) before and after its spread into Western Europe and Asia, and iii) assessing how this risk varies with the US airport, country and month. These results may inform decisions related to the design of ASFV surveillance strategies in the US.

## Results

The mean annual probability that ASFV-contaminated PSPAP arrives in a US airport prior to customs inspection) was estimated at 0.21 (95% CI 0.19–0.76). The mean annual probability that ASFV-contaminated PSPAP enters the US (after customs inspection) was estimated at 0.11 (95% CI 0.01–0.50), which is a 183.33% higher than the risk of introduction that we estimated prior to the spread of the disease through Europe and Asia in 2018 and 2019. Our latest estimate suggests that ASFV may evade customs controls and be introduced into the US at least once every 9 years on average, with the lower boundary of the 95% confidence interval corresponding to once every 2 years. China, Hong Kong, the Russian Federation, and Poland account for 97% of the risk, with all other countries contributing <1% of the risk (Table 1).

**Table 1:** Annual risk (probability) of African Swine Fever Virus (ASFV) introduction into the US through prohibited swine products carried in air passenger luggage (PSPAP) per administrative unit and continent of origin.

Mean annual risk per continent*	Administrative Unit	% total risk	Mean annual risk
Asia [0.076]	China	38.35%	4.28 × 10^−2^
	Hong Kong	29.33%	3.28 × 10^−2^
	Others	0.36%	4 × 10^−4^
Europe [0.034]	The Russian Federation	26.92%	3 × 10^−2^
	Poland	2.43%	2.71 × 10^−3^
	Others	1.17%	1.29 × 10^−3^
Africa [0.002]	—	1.44%	1.6 × 10^−3^

^*^Mean annual risk per continent was calculated by using the mean annual risk of the different countries belonging to such continent.

**Figure 1:**
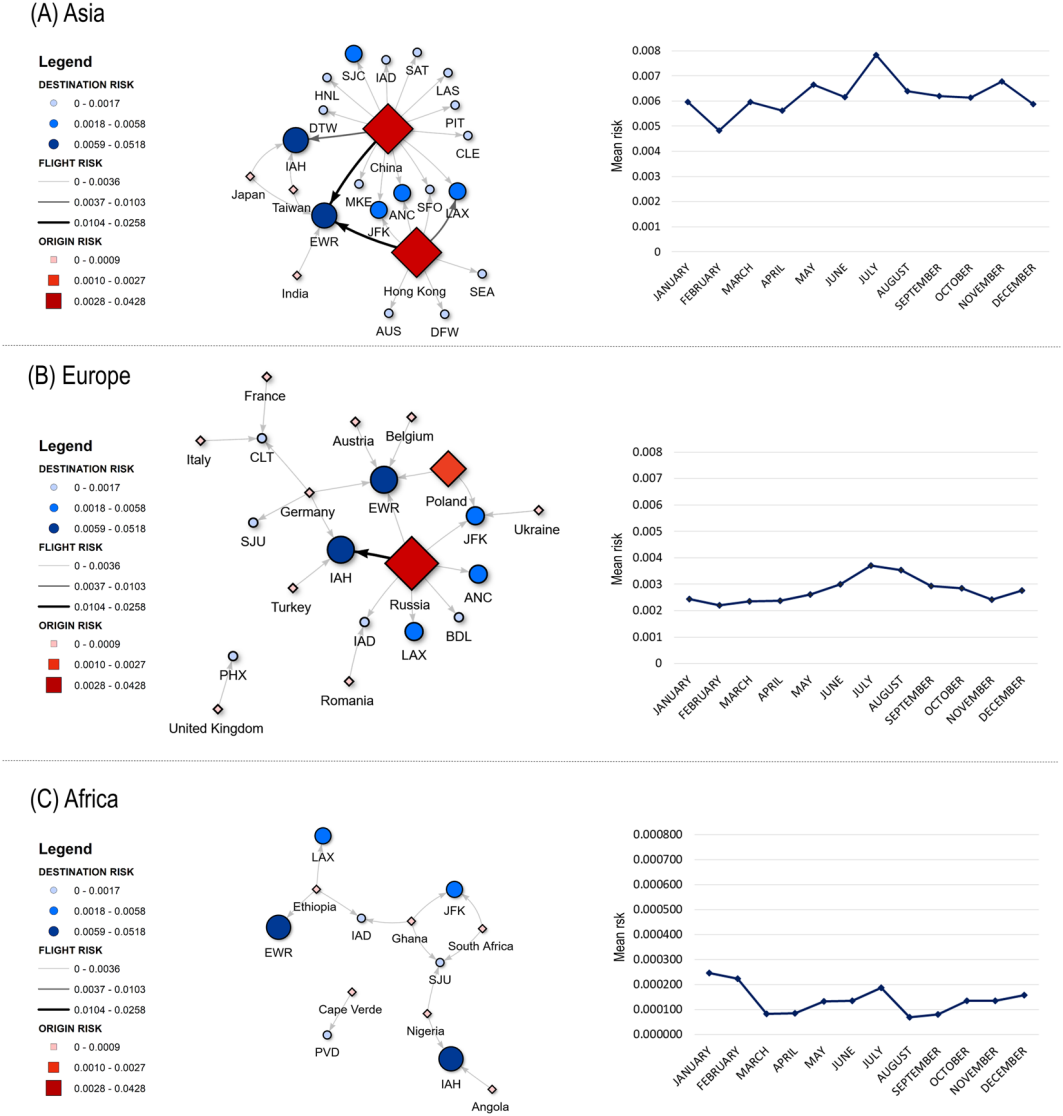
Mean annual and monthly risk (probability) of African Swine Fever Virus (ASFV) introduction per country of origin, destination airport in US and flight. (**A**) Asia; (**B**) Europe; and (**C**) Africa. Airports are represented by their IATA codes; full names are shown in Supplementary Table S1.

Five airports accounted for >90% of the risk: Newark-New Jersey (46.38%), George Bush-Houston-Texas (32.71%), Los Angeles-California (5.18%), John F. Kennedy-New York (5.04%) and San Jose-California (2.87%). Regardless of the country or region of origin, risk was higher during the summer, particularly July (Fig. 1).

**Figure 2:**
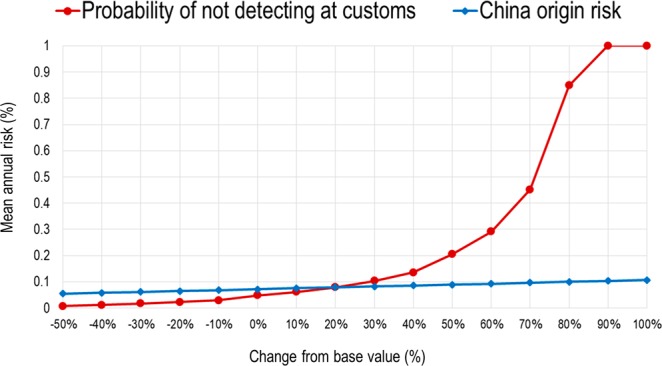
Advanced sensitivity analysis for the risk (probability) of African Swine Fever Virus (ASFV) introduction into the US through prohibited swine products carried in air passenger luggage (PSPAP). Graphs plot the percentage of change in the probability of non-detection of PSPAP at customs inspection (red line), the probability of infection in China (blue line) and the impact on the mean annual risk.

The primary output of the model (the probability that ASFV-contaminated PSPAP arrives in the US after customs inspection) was influenced most by the probability of infection in China (ρ_i_ = 0.61) and the probability of not detecting PSPAP at customs (ρ_i_ = 0.72). Consistent with these results, the probability of not detecting PSPAP at customs accounted for 38.17% of the variance in probability of PSPAP arrival in the US, while the probability of infection in China accounted for 19.53% of the variance. Sensitivity analysis showed that these two factors together doubled the annual risk of PSPAP arrival in the US, independently of other factors. Figure 2 illustrates the results of the advanced sensitivity analysis, showing how these two factors influenced the final annual risk from a reduction of 50% of their base values to an increase of 100%.

## Discussion

In response to the spread of ASF in Western Europe and Asia in 2018 and 2019, disease-free countries have enforced strategies, control measures, and biosecurity protocols to protect their susceptible populations against the disease. Many believe that products illegally introduced through passenger luggage constitute a substantial source of risk for spread of FADs^21–28^ such as ASF. For example, the UK Department of Environment, Food and Rural Affairs (DEFRA) has estimated that ASFV is likely (with moderate uncertainty) to be introduced into the UK from EU member states through contaminated products^28^. Consequently, many countries have strengthened the control of air passenger luggage at points of entry^29–31^. Perhaps in part as a result of these efforts, ASFV-contaminated pork products, such as sausages or dried pork, have been detected several times at ports and airports in Taiwan, South Korea, Japan, Thailand, Australia, Philippines and United Kingdom in 2018–2019 (Supplementary Table S2)^13–19^. The infectivity of these products is unknown, except for products confiscated in Japan, where some products were confirmed to contain infective virus^32,33^. Most of the products confiscated at these airports originated from China, where meat and pork products have been found to contain ASFV (Table S2). Consequently, Chinese authorities have required companies to trace the source of contaminated raw materials and prevent contaminated pork raw materials from entering the food chain^7^. The effectiveness of those preventive measures has yet to be measured.

The results here suggest a high risk that ASFV can reach US airports as PSPAP prior to customs control. In this study, all PSPAP were assumed to be contaminated with ASFV according to the principle of maximum risk, what might have led to overestimate the probability of introduction. Nevertheless, the viral genome has been detected in confiscated pork at many international airports, but whether the virus has entered US airports is unclear since pork seized in that country is not tested for ASFV. These results, along with the estimated 183.33% increase in risk of ASFV introduction into the country following spread of the disease in Asia and Europe, underscore the importance of prevention and detection measures at US airports. Most risk seems to concentrate on flights from China, Hong Kong, the Russian Federation and Poland, in contrast to earlier estimates that attributed most risk to flights from Africa^20^. Indeed, the much higher cost of pork and much smaller pork production in the US than in China create an incentive for large-scale pork smuggling into the US. In March-2019, an illegal shipment of 1 million pounds of pork from China was seized at Newark airport^34^.

Noteworthy, results were highly sensitive to the probability of non-detection of illegal products at airport. The probability of non-detection at customs was previously estimated by other authors^20,35,36^. This probability was estimated as a unique value for the whole US. This assumption might have led to underestimate or overestimate the capacity of detection of some airports as the CBP resources and volume of passengers could considerably differ between airports. Because of the influence of that parameter in the model outputs, and because of the challenges associated with the accurate prediction of its true value, it is possible that the risk of ASFV introduction into the country would actually be lower or higher than the values estimated here. Therefore, it would be beneficial for the model to include actual information on the efficacy of control disaggregated by airport.

The US swine industry is one of the most industrialized in the world, and biosecurity measures are strictly enforced as the final line of defense to protect domestic pigs from ASFV incursions. Much of the concern related to the ASFV introduction through air passenger luggage is linked to the possibility that contaminated products may be disposed of outside the airport control zone, where it may infect feral pigs^28,37^. The population of feral pigs has steadily increased in the US, likely due to their flexibility in adapting to a variety of habitats as well as a lack of natural predators. Feral swine are present in at least 35 US states, giving rise to a population of >6 million animals^38^. ASF spreads through populations of wild boar and feral pigs, so ASFV-contaminated products disposed of outside the airport may pose a risk to the domestic pig population in the US.

Summer months, and particularly July, accounted for most of the risk in our assessment. Summer is the time of the year when most tourists visit the US; in July 2017, for example, almost 23 million people flew into the US^39^. The agricultural inspection process at US airports consists of primary activities (inspection of customs documents, interviewing of passengers, and searches by agriculture canine teams for agricultural products in the baggage area) and secondary activities (interviewing and luggage inspection). The large volume of passengers arriving every year into the US may compromise the efficacy of these activities. Targeted surveillance is a key strategy to increase effectiveness of prevention measures when resources are limited. Results here may be used to inform recommendations regarding how to strengthen surveillance activities in the US. Our results suggest that measures to detect ASFV early and prevent disease may be selectively targeted or prioritized to five airports, especially during the summer.

A limitation of the work here is that the probability of carrying pork products was assumed to be equal across countries and regions of origin. However, cultural and religious factors can influence passengers’ behavior^24,27^. Future studies should generate data on this point in order to incorporate it into the model. Another limitation of our study is that we had no information on connecting flights prior to arrival in the US, so we could not estimate risk from the point of departure. This means that our model may underestimate the risk of ASFV arrival due to the lack of information on the number of passengers from affected countries with no direct flights to the US. These passengers (*i*.*e*. passengers from Estonia, Belarus and Moldova, among others) would have layover at major hubs such as Amsterdam, London Heathrow, Frankfurt, or Paris-Charles de Gaulle. As a consequence of such absence of information, the risk estimated for countries with no direct flights into the US, and most importantly, the risk estimated for countries with major airport hubs, may have been underestimated here. Moreover, if data on connecting flights were available, the probability of detection of PSPAP at connecting hubs and data on confiscations at such customs control should be also taken into consideration to avoid underestimating or overestimating the final risk.

In conclusion, results suggest that the risk of ASFV introduction into the US through smuggling of pork through air passenger luggage has increased substantially since the disease spread into regions of Asia and Europe in 2018 and 2019. Most of the risk appears to come from China (38.35%), Hong Kong (29.32%), the Russian Federation (26.92%) and Poland (2.43%). The majority of risk concentrates in five US airports and is higher in the summer. These results will help to inform decisions related to design of ASFV surveillance strategies in the US.

## Methods

The probability of ASFV introduction into the US through PSPAP (defined as output) was assessed using a quantitative stochastic model. This probability (also named as risk) was estimated for each of 128 countries or regions of origin, for each of 87 US airports, and flights. The probability was also estimated across all US airports. The level of risk was assessed monthly and annually, using a model adapted from an earlier one^20^. The risk model was developed in @RISK 7.6 (Palisade Corporation, Newfield, NY, USA) on Microsoft Excel 2007® and run for 10,000 iterations using a Monte-Carlo sampling method.

The primary output of the model was the probability that ASFV contaminated pork reaches the US (after customs inspection), based on data after the virus already spread through Europe and China in 2018 and 2019. This probability was compared to that obtained with data from before that expansion^20^. As an additional output, the probability that ASFV-contaminated products arrive at US airports (before customs inspection) was estimated by computing the probability of ASFV introduction without considering the probability of non-detection at customs.

Briefly, the risk was calculated using two main input datasets (defined as inputs), namely, i) data on the number of PSPAPs confiscated at US airports by Customs and Border Protection from January 2010 to March 2016 at US airports, from the Animal and Plant Health Inspection Service of the US Department of Agriculture (obtained from USDA/APHIS dataset); and ii) information on the number of air passengers arriving in the US via international commercial flights from January 2010 through May 2018.

The probability of ASFV introduction via PSPAP was modelled as a binomial process of the form

$$P(x\ge 1)=\sum 1-1{({{\rm{Pi}}}_{{\rm{o}}})}^{{\rm{Nodm}}}$$

where Pi_o_ is the estimated probability that at least 1 kg of PSPAP from each origin country or region is contaminated with ASFV. This probability was assumed to be equivalent to at least one domestic pig infected with ASFV. The probability of infection in each country or region of origin (n = 128) was estimated based on disease information obtained from the OIE-WAHIS database^5^ from the date when the disease was introduced through February 7, 2019. For the present study, countries or regions of origin were classified as (i) “high risk” if ASF was present and/or suspected in domestic pig, (ii) “medium risk” if ASF was present only in wild boar and/or the country or region bordered an infected country or region; or (iii) “low risk” in all other cases.

The probability of ASFV infection in “high risk” countries or regions was estimated by taking into account the potential number of non-reported infected pigs and the number of pigs slaughtered monthly in the country or region, following an approach described elsewhere^40^. This probability was estimated following a beta distribution with parameters α_1_ and α_2,_ where α_1_ = Ni_om_ × Mod Prop-S_m_ + 1 and α_2_ = NS_om_ − Ni_om_ × Mod Prop-S_m_ + 1. Ni_om_ denotes the number of infected and non-reported domestic pigs in each origin country *o* per month *m*. The number of infected and non-reported pigs per *o* and *m* was estimated by multiplying the mean census on affected farms in *o*, the mean prevalence on affected farms, the mean number of outbreaks per month, and the duration of ASFV infection in months; this multiplicative product was divided by the probability of notification underreporting^36^ and by the time in months since disease introduction in *o*. If the mean size of affected farms was unavailable, the mean pig farm size in *o* was obtained from the FAOSTAT database^1^. The estimated number of slaughtered, infected, and non-reported pigs was calculated by multiplying the estimated number of infected and non-reported domestic pigs by the proportion of the annual pig census slaughtered per month in infected countries (Mod Prop-S_m_). NS_om_, which represents the number of pigs slaughtered for meat production per *o* and *m*, was calculated by multiplying the pig census per *o* by the proportion of annual pig census slaughtered per *m* (Prop-S_m_). The probability of infection in countries belonging to “medium risk” and “low risk” countries or regions was calculated following the approach of Jurado *et al*.^20^ where the probability of an outbreak was multiplied by the average size of an outbreak, the duration of the infection, the probability of an outbreak not being detected and the proportion of pigs slaughtered per month. The final probability of outbreak occurrence was considered to be 10 times higher for “medium risk” than “low risk” countries or regions^20^.

Data on animal populations in countries or regions of origin for the present model came from 2016, whereas our model of risk from before ASFV spread to Europe and Asia relied on data from 2010. Since data on outbreaks and pig populations are unavailable for Guinea, Liberia and Ethiopia, we defined the probability of infection in these countries as the median of the probability for all other African countries. The probability of infection in Hong Kong was assumed to be the same as China, which supplies 94% of pork products consumed in Hong Kong^41^.

N^odm^ is the estimated volume (kg) of PSPAP introduced into each US airport *d* from each *o* per *m*. Briefly, N^odm^ was estimated by multiplying the estimated number of kg of PSPAP confiscated from each *o* per *d* per *m* by the probability of PSPAP non-detection at customs (Supplementary Table S3). All PSPAP were assumed to be contaminated with ASFV based on the principle of maximum risk, especially since the database did not record whether the pork product was raw or treated chemically or thermally. The probability of non-detection at customs was assumed to be the same for all US airports and modelled following the approach of Jurado *et al*.^20^, while taking into account improvements in detection estimated by previous studies^35,36^. Supplementary Table S3 lists formulas and sources of information used to estimate both parameters.

The input parameters of the model that most heavily influenced the output probability of ASFV entry into the US were identified by conducting two-step sensitivity analysis. First, inputs that had Spearman correlation coefficients (ρ_i_) > 0.5 and that contributed >25% to the variance of the output were selected for analysis in detail involving 1,000 iterations for each of 16 scenarios generated by changing the base value of the parameter in consecutive steps, from a minimum reduction of −50% value to a maximum increase of 100%.

Flights accounting for 99% of the annual risk for US airports were selected to build networks per continent (e.g. flights representing <1% of the total risk were not displayed for clarity of the net), and depicted showing mean annual risk and the associated 95% confidence interval (CI) per country of origin, US destination airport, and flight level. Risks at the three levels were categorized using Jenks’ natural break classification method^42^. Nets were built using visNetwork package^43^ implemented in R software^44^. Results were mapped using ArcMap 10.3 (ESRI®).

## Supplementary information

Table S1-S3
