# Supplementary material for: Risk of African swine fever virus introduction into the United States through smuggling of pork in air passenger luggage
Source: Sci Rep. 2019 Oct 8;9:14423. doi: 10.1038/s41598-019-50403-w (PMC6783460; doi:10.1038/s41598-019-50403-w)
Supplement: Supplementary file 1 — Table S1-S3 [file 41598_2019_50403_MOESM1_ESM.pdf]

Risk of African swine fever virus introduction into the United States through smuggling of pork in air passenger luggage

Cristina Jurado[1, 2]*, Lina Mur[2], María Sol Pérez Aguirreburualde[3], Estefanía Cadenas-Fernández[1], Beatriz Martínez-López[4], José Manuel Sánchez-Vizcaíno[1], Andrés Perez[3]

[1] VISAVET Health Surveillance Centre and Animal Health Department, Complutense University of Madrid, Madrid, Spain

[2] Department of Diagnosis Medicine/Pathobiology, College of Veterinary Medicine, Kansas State University, Kansas State University, Manhattan, Kansas, USA

[3] Center for Animal Health and Food Safety, College of Veterinary Medicine, University of Minnesota, Saint Paul, Minnesota, USA

[4] Center for Animal Disease Modeling and Surveillance; and Department of Medicine & Epidemiology, School of Veterinary Medicine, University of California, Davis, California, USA

*** Correspondence:**
Cristina Jurado
cjdiaz@ucm.es

Table S1. List of airports including their IATA code, name, city and state location.

Airport IATA code	Airport Name	City name	State
ALB	Albany International Airport	Albany	New York
ANC	Ted Stevens Anchorage International Airport	Anchorage	Alaska
ATL	Hartsfield–Jackson Atlanta International Airport	Atlanta	Georgia
AUS	Austin–Bergstrom International Airport	Austin	Texas
BDL	Bradley International Airport	Windsor Locks	Connecticut
BFI	Boeing Field	Seattle	Washington
BIF	Biggs Army Airfield	El Paso	Texas
BLI	Bellingham International Airport	Bellingham	Washington
BNA	Nashville International Airport	Nashville	Tennessee
BOS	Logan International Airport	Boston	Massachusetts
BQK	Brunswick Golden Isles Airport	Brunswick	Georgia
BQN	Rafael Hernández Airport	Aguadilla	Puerto Rico
BRO	Brownsville/South Padre Island International Airport	Brownsville	Texas
BUF	Buffalo Niagara International Airport	Buffalo	New York
BWI	Baltimore–Washington International Airport	Baltimore	Maryland
CHS	Charleston International Airport	Charleston	South Carolina
CLE	Cleveland Hopkins International Airport	Cleveland	Ohio
CLT	Charlotte Douglas International Airport	Charlotte	North Carolina
CMH	John Glenn Columbus International Airport	Columbus	Ohio
CVG	Cincinnati/Northern Kentucky International Airport	Hebron	Kentucky
DEN	Denver International Airport	Denver	Colorado
DFW	Dallas/Fort Worth International Airport	Dallas	Texas
DOV	Dover Air Force Base	Dover	Delaware
DTW	Detroit Metropolitan Airport	Detroit	Michigan
EFD	Ellington Airport	Houston	Texas
EWR	Newark Liberty International Airport	Newark	New Jersey
EYW	Key West International Airport	Key West	Florida
FAI	Fairbanks International Airport	Fairbanks	Alaska
FAT	Fresno Yosemite International Airport	Fresno	California
FLL	Fort Lauderdale–Hollywood International Airport	Fort Lauderdale	Florida
HNL	Daniel K. Inouye International Airport	Honolulu	Hawaii
HOU	William P. Hobby Airport	Houston	Texas
HSV	Huntsville International Airport	Huntsville	Alabama
IAD	Washington Dulles International Airport	Dulles	Virginia
IAH	George Bush Intercontinental Airport	Houston	Texas
IND	Indianapolis International Airport	Indianapolis	Indiana
INL	Falls International Airport	International Falls	Minnesota

JFK	John F. Kennedy International Airport	Queens	New York
KOA	Kona International Airport	Kailua-Kona	Hawaii
LAS	McCarran International Airport	Las Vegas	Nevada
LAX	Los Angeles International Airport	Los Angeles	California
LRD	Laredo International Airport	Laredo	Texas
MCI	Kansas City International Airport	Kansas City	Missouri
MCO	Orlando International Airport	Orlando	Florida
MDW	Chicago Midway International Airport	Chicago	Illinois
MEM	Memphis International Airport	Memphis	Tennessee
MFE	McAllen Miller International Airport	McAllen	Texas
MIA	Miami International Airport	Miami	Florida
MKE	General Mitchell International Airport	Milwaukee	Wisconsin
MSP	Minneapolis–Saint Paul International Airport	Minneapolis	Minnesota
MSY	Louis Armstrong New Orleans International Airport	Kenner	Louisiana
NGU	Norfolk Naval Station	Norfolk	Virginia
NYL	Marine Corps Air Station Yuma	Yuma	Arizona
OAK	Oakland International Airport	Oakland	California
ONT	Ontario International Airport	Ontario	California
ORD	O'Hare International Airport	Chicago	Illinois
PBI	Palm Beach International Airport	West Palm Beach	Florida
PDX	Portland International Airport	Portland	Oregon
PHL	Philadelphia International Airport	Philadelphia	Pennsylvania
PHX	Phoenix Sky Harbor International Airport	Phoenix	Arizona
PIT	Pittsburgh International Airport	Pittsburgh	Pennsylvania
PNS	Pensacola International Airport	Pensacola	Florida
PSE	Mercedita Airport	Ponce	Puerto Rico
PVD	T. F. Green Airport	Warwick	Rhode Island
PWM	Portland International Jetport	Portland	Maine
RDU	Raleigh–Durham International Airport	Raleigh	North Carolina
RIC	Richmond International Airport	Richmond	Virginia
RNO	Reno–Tahoe International Airport	Reno	Nevada
ROC	Greater Rochester International Airport	Rochester	New York
RSW	Southwest Florida International Airport	Fort Myers	Florida
SAN	San Diego International Airport	San Diego	California
SAT	San Antonio International Airport	San Antonio	Texas
SDF	Louisville International Airport	Louisville	Kentucky
SEA	Seattle–Tacoma International Airport	Seattle	Washington
SFB	Orlando Sanford International Airport	Sanford CBP	Florida
SFO	San Francisco International Airport	San Francisco	California
SJC	San Jose International Airport	San Jose	California
SJU	San Juan Luis Muñoz Marín airport	San Juan	Puerto Rico

SLC	Salt Lake City International Airport	Salt Lake City	Utah
SMF	Sacramento International Airport	Sacramento	California
SNA	John Wayne Airport	Santa Ana	California
STL	St. Louis Lambert International Airport	Saint Louis	Missouri
STT	Cyril E. King Airport	Saint Thomas	United States Virgin Islands
STX	Henry E. Rohlsen Airport	Saint Croix	United States Virgin Islands
SYR	Syracuse Hancock International Airport	Syracuse	New York
TPA	Tampa International Airport	Tampa	Florida
TUS	Tucson International Airport	Tucson	Arizona

Table S2. * Proportion of seized product detected positive to African swine fever virus (ASFV).

**Apparent frequency (monthly based).

**Country**	**Date published**	**Event**	**Proportion ***	**Frequency ****	**Source**
South Korea	08-26-2018	ASFV detected in dumplings and sausage declared by a South Korean tourist returning from Shenyang province	---	---	Link
Japan	10-22-2018	Detection of ASFV in packed pork sausage brought by a passenger from Beijing to Shin-Chitose airport in Hokkaido	---	---	Link
Taiwan	10-31-2018	Since late August 2018, 928 products have been seized and test for ASFV, 20 samples have been found to be positive	2.1%	2.86 events per month	Link
Thailand	01-16-2019	ASF virus detected in pork products at Phuket Airport	---	---	Link
Australia	01-17-2019	6 of 152 pork products seized over a period of two weeks were contaminated with ASFV	3.2%	10 events per month	Link
Philippines	06-14-2019	Canned pork products seized at the Clark International Airport in Pampanga brought in from Hong Kong in March resulted positive for ASFV by PCR	---	---	Link
United Kingdom	07-11-2019	In July, airport authorities in Northern Ireland seized over 300 kg of illegal meat products. A sample of these	---	---	Link

		seizures resulted positive for ASFV by PCR.			

Table S3. Description of input parameters, data sources and probabilities used to estimate the probability of introduction of prohibited swine products carried in air passenger luggage (PSPAP) contaminated with African Swine Fever Virus (ASFV) into US airports, stratified by month, country of origin and destination airport.

Notation	Definition	Parametrization	Values	Source/ Reference
PAS	Number of air passengers arriving in commercial flights in the US per origin (*o*), destination airport (*d*) and month (*m*)	Normal (μ, σ)	NA	T-100 international segment database (all carriers)[45]
VL/PAS_o	Volume in Kilograms (kg) of luggage per air passenger, per *o*	Pert (min, most likely, max)	NA	Checked baggage allowance of United Air Lines Inc., Delta Air Lines Inc., and American Airlines Inc[46-48]
VL_{odm}	Total kg of luggage per *o*, *d* and *m*		$PAS_{odm} * VL/PAS_o$	
Prop-VL_{odm}	Proportion of kg of luggage from each *o* arriving at each *d*, per *m*	Normal (μ, σ)	$VL_{odm} / \sum_{d}^{i} VL_{odm}$	
$PSPAP_{dm}$	Median number of PSPAP confiscations per *d* and *m*	Median		Agricultural Activity Work Accomplishment Database, Animal and Plant Health Inspection Service, US Department of Agriculture
VL/PSPAP	Individual volume (kg) of each PSPAP	Pert (min, most likely, max)	Pert (1.5, 3.25, 5)	[20]

$\sum V_{PSPAP}$	Total volume (kg) of PSPAP confiscated per *d*, per *m*		$PSPAP_{dm}$ * VL/PSPAP	
CON-PSPAP $_{odm}$	kg of PSPAP confiscated at customs and border protection (CBP) control per *o, d* and *m*		Prop VL_{odm} *$\sum V_{PSPAP}$	
$P_{non\text{-}det}$	Probability of PSPAP non-detection at CBP controls	Triang (min, most likely, max)	Triang (0.2, 0.5, 0.9)	[20]
N_{odm}	kg of PSPAP escaping CBP controls, introduced into the US per *odm*		CON-$PSPAP_{odm}$ * $P_{non\text{-}det}$ / (1 - $P_{non\text{-}det}$)	
N_o	Pig census in origin country (*o)*	NA	Data from year 2016	[1,5]
Prop-S_m	Proportion of annual pig census slaughtered per month (*m*)	Pert (min, most likely, max)	Pert (0.1, 0.18, 0.25)	[49]
NS_{om}	Number of pigs slaughtered for meat production in country *o* per month *m*	Pert (min, most likely, max)	N_o * Prop-S_m	
P_U	Probability of notification underreporting	Pert (min, most likely, max)	Pert (0.2, 0.4, 0.6)	[50]
D	Duration of ASFV infection (months)	Pert (min, most likely, max)	Pert (3, 11, 30)/30	[20]
Ni_{om}	Number of ASFV non-reported infected pigs in infected *o* and *m*		(Mean pig census on affected farms * mean prevalence on affected farms * number of outbreaks * d) / (P_U * time since disease presence in months)	[1,5]
Mod Prop-S_m	Modified proportion of annual pig census slaughtered per month (m)	Uniform (min, max)	min = Prop-S_m<br>max = 1	[20]
$Pi_{High\ Risk\ o}$	Monthly probability of ASFV infection in pigs slaughtered in *o*	Beta (α_1, α_2)	α_1 = Ni_{om} * Mod Prop-S_m + 1<br>α_2 = NS_{om} – Ni_{om} * Mod Prop-S_m + 1	

$Prop_{AF}$	Proportion of pig population affected by disease outbreak	Pert (min, most likely, max)	Pert (0, 0.002, 0.02)	50
$Po_{Medium\ Risk\ o}$	Probability of ASF outbreak in "medium risk" countries or regions	Pert (min, most likely, max)	Pert (0, 0.0022, 0.022)	20
$Pi_{Medium\ Risk\ o}$	Probability of ASFV infection in pigs slaughtered in *o* and *m*	Pert (min, most likely, max)	$Po_{Medium\ Risk\ o} * Prop_{AF} * d * P_U$	
$Po_{Low\ Risk\ o}$	Probability of ASF outbreak in "low risk" countries or regions	Pert (min, most likely, max)	Pert (0, 0.00022, 0.0022)	20
$Pi_{Low\ Risk\ o}$	Probability of ASF outbreak in "low risk" countries or regions	Pert (min, most likely, max)	$Po_{Low\ Risk\ o} * Prop_{AF} * d * P_U$	

Supplementary references

45 United States Department of Transportation. Bureau of transportation statistics. T-100 international segment (all carriers). < https://www.transtats.bts.gov/> (USDT, 2017).

46 American Airlines Inc. Baggage regions. <https://www.aa.com/i18n/travel-info/baggage/baggage-regions.jsp>. (2017).

47 Delta Air Lines Inc. Baggage and travel fees. <http://www.delta.com/content/www/en_US/travelingwith>. (2017).

48 United Air Lines Inc. (2017). Checked baggage.< https://www.united.com/CMS/en-US/travel/Pages/CheckedBaggage.aspx>. (2017).

49 European Commission Statistics. <http://ec.europa.eu/eurostat>. (EUROSTAT, 2017)

50 Veterinary Laboratories Agency. Risk Assessment for the Import of Contaminated Meat and Meat Products into Great Britain and the Subsequent Exposure of GB Livestock. <http://webarchive.nationalarchives.gov.uk/20061209024841/http://www.defra.gov.uk/animalh/illegali/reports/index.htm>. (VLA, 2004).
